# Magneto-Mechanically Triggered Thick Films for Drug Delivery Micropumps

**DOI:** 10.3390/nano12203598

**Published:** 2022-10-13

**Authors:** Georgiana Dolete, Cristina Chircov, Ludmila Motelica, Denisa Ficai, Ovidiu-Cristian Oprea, Marin Gheorghe, Anton Ficai, Ecaterina Andronescu

**Affiliations:** 1 Department of Science and Engineering of Oxide Materials and Nanomaterials, Faculty of Chemical Engineering and Biotechnologies, University Politehnica of Bucharest, Gh. Polizu 1-7, 011061 Bucharest, Romania; 2 National Center for Micro and Nanomaterials, University Politehnica of Bucharest, Splaiul Independentei 313, 060042 Bucharest, Romania; 3 Department of Inorganic Chemistry, Physical Chemistry, and Electrochemistry, Faculty of Chemical Engineering and Biotechnologies, University Politehnica of Bucharest, Gh. Polizu 1-7, 011061 Bucharest, Romania; 4 Academy of Romanian Scientists, Ilfov Street 3, 050044 Bucharest, Romania; 5 SC NANOMEMS SRL, George Coșbuc 9, 505400 Râșnov, Romania; 6 Center for Technological Electronics and Interconnection Techniques, University Politehnica of Bucharest, Bulevardul Iuliu Maniu, 061071 Bucharest, Romania

**Keywords:** magnetic polymer thick films, magneto-mechanical triggering, drug delivery, BioMEMs

## Abstract

Given the demanding use of controlled drug delivery systems, our attention was focused on developing a magnetic film that can be triggered in the presence of a magnetic field for both drug delivery and the actuating mechanism in micropump biomedical microelectromechanical systems (BioMEMS). Magnetic alginate films were fabricated in three steps: the co-precipitation of iron salts in an alkaline environment to obtain magnetite nanoparticles (Fe_3_O_4_), the mixing of the obtained nanoparticles with a sodium alginate solution containing glycerol as a plasticizer and folic acid as an active substance, and finally the casting of the final solution in a Petri dish followed by cross-linking with calcium chloride solution. Magnetite nanoparticles were incorporated in the alginate matrix because of the well-established biocompatibility of both materials, a property that would make the film convenient for implantable BioMEMS devices. The obtained film was analyzed in terms of its magnetic, structural, and morphological properties. To demonstrate the hypothesis that the magnetic field can be used to trigger drug release from the films, we studied the release profile in an aqueous medium (pH = 8) using a NdFeB magnet as a triggering factor.

## 1. Introduction

A drug delivery system (DDS) is defined as a material or technology capable of introducing a therapeutic substance into the body, so that the efficiency and safety of that substance are enhanced, by controlling its release in terms of dose, time, or place of action [1]. The supreme goal for drug delivery research is focused on developing therapeutically viable formulations that can aid patients in the treatment of various conditions. Advances in nanotechnologies have become a spotlight in expanding the field of DDSs [2], and intensive effort has been made in developing different approaches. A broad classification suggests the use of organic and inorganic nanomaterials. DDSs can be divided depending on the transport/targeting/release mechanism using different strategies such as stimuli-responsive materials, porous materials, robots, and microelectromechanical system (MEMS) devices. Inorganic nanomaterials are modified so that they meet the requirements for their use in the systemic circulation to develop controlled and targeted therapies using a wide variety of small or large molecules [3]. Iron oxide nanoparticles, for instance, are widely explored in cancer treatment as drug delivery carriers [4,5,6] as well as in hyperthermia treatment [7,8]. Moreover, combining inorganic nanoparticles with stimuli-responsive materials led to a great improvement in cancer treatment by enhancing the drug cellular uptake and cytotoxicity of cancer cells. The mechanism by which these more controlled and targeted releases are possible is based on physical or chemical changes induced in the system by exposure to external stimuli or triggers [9]. So far, much research has been conducted on materials capable of responding to temperature [10,11,12], pH [13], ultrasound [14,15], light [16,17], electrical [18], or magnetic stimuli. Kost et al. [19] were the first to use an external alternative magnetic field to generate the pulsatile release of insulin from an ethylene acetate copolymer composite with embedded magnets implanted in diabetic rats. Their study suggested a much faster release of the drug was possible by applying an external magnet in the proximity of the material. A stimulus-responsive drug delivery system based on titania nanotubes supplied with magnetic nanoparticles at the bottom of the nanotubes showed a complete release of indomethacin from polymeric micelles in a maximum time period of 1.5 h when applying a magnetic field [20]. Furthermore, dopamine levels were observed to increase by 24–33% in response to applying an electromagnetic field to alginate/magnetite beads [21]. Several reviews focus on cancer, cardiovascular, and neurodegenerative applications in the use of magneto-responsive materials for drug transport [22,23,24].

The newest strategy, which unequivocally evolved from advancements in the miniaturization of the microelectromechanical components of nanotechnologies, is the use of MEMS devices. Since the discovery of MEMS, chemical and biological analyses, including molecular separation or environmental monitoring [25,26], as well as applications related to the pharmaceutical industry, have improved considerably [27]. MEMS used in biological applications is referred to as BioMEMS [28], and they are already being spun out into commercial applications using different types of devices such as micro-pumps [29], micro-robots [30,31], biosensors [32], or microneedles [33]. There exists a considerable body of literature exploiting drug delivery micropumps using different types of actuation mechanisms such as piezoelectric [34,35,36], electrostatic [37], or electromagnetic [38] mechanisms. Pirmoradi et al. [39,40] investigated micro-pumps based on a magnetic membrane provided with a centered laser-drilled aperture that could trigger drug release from a reservoir in the presence of a magnetic field. According to their studies, 3.4 ng of docetaxel could be released every minute in the presence of a 0.25 T magnetic field [40].

As far as we know, there is no previous research using a magnetic stimulus to enhance drug release via a magneto-mechanical mechanism. Starting from this idea, the assumption behind our premise is that the application of an external magnetic field on the surface of a fixed film or membrane induces a deformation, followed by a return to its original position after removing the magnetic field. As a result of this actuation, we assumed that the film/membrane would tend to release faster molecules incorporated into the film, being driven primarily by a mechanic release promoted by the magnetic stimulus. This work aimed to prove the feasibility of using magnetic fields as a triggering factor for further use as integrated components in drug delivery systems based on micropumps. Sodium alginate was selected as the polymeric matrix due to the alginate structure that allows for the incorporation of different types of molecules and due to its well-studied biocompatibility inducing adhesion, proliferation, and differentiation of several cells [41,42]. The system could further be used for implantable devices based on these properties. Folic acid is a vitamin that is primarily used in the targeted administration of chemotherapeutics due to the presence of folate receptors on the surface of most cancer cells and in this context acts as a model drug. The presence of these receptors induces an affinity of cancer cells for systems that possess the conjugated form of folic acid, establishing a bond and facilitating the internalization of antitumor agents [43]. In addition to its established role in the development of controlled release systems in cancer treatment, we chose folic acid as the molecule of interest for demonstrating our hypothesis due to its low solubility in neutral pH, thus avoiding its release during crosslinking in CaCl_2_ solution.

## 2. Materials and Methods

### 2.1. Materials

All chemicals used in the study were analytical grade and used without further purification. Ferric chloride (FeCl_3_), ferrous sulfate heptahydrate (FeSO_4_·7H_2_O), sodium hydroxide (NaOH), sodium alginate, and folic acid (FA) were purchased from Sigma-Aldrich Merck (Darmstadt, Germany). Glycerin was purchased from Silal Trading SRL (Silal Trading SRL, Bucharest, Romania).

### 2.2. Methods

#### 2.2.1. Synthesis of Magnetic Nanoparticles

First, we obtained the magnetic nanoparticles through a modified co-precipitation method, using a low magnetic field, similar to that in our previous paper [44]. Precisely, an aqueous solution of the ferrous and ferric salts (molar ratio Fe^2+^:Fe^3+^ = 1:2) was added dropwise to a 10% NaOH solution. Solutions were stirred at 250 rpm using a BIOSAN MM-1000 mechanical stirrer and a permanent NdFeB magnet was placed under the beaker.

#### 2.2.2. Alginate Magnetic Thick Films

A solution of 2% sodium alginate (*w*/*v*) was obtained by dissolving an adequate quantity of sodium alginate in double distilled water. According to previous studies, the flexibility of alginate films is improved by the addition of different plasticizers such as polyols [45]. Glycerol was added to the polymeric solution so that a final concentration of 10% glycerol (*v*/*v*) was reached. The alginate/glycerol mixture was mechanically stirred for 1 h at room temperature. A total of 0.3 g of folic acid (FA) was added to 15 mL of the previously obtained mixture and left under vigorous mechanical stirring for another hour. Lastly, we added the magnetic nanoparticles in a 0.5:1 ratio to the sodium alginate mass. To improve the homogenization of the magnetic particles in the polymeric matrix, the mixture was mechanically stirred for 3 h at 300 rpm. The final suspension, containing sodium alginate, glycerol, FA, and Fe_3_O_4_, was cast in a Petri dish (95 mm in diameter) and placed in an oven at 40 °C overnight. The dried composite membrane was then cross-linked for 15 min with CaCl_2_ 2% (*w*/*v*), resulting in the magnetic film labeled as 10G/Alg/FA/Fe_3_O_4_. The cross-linking solution did not present any traces of folic acid, so we considered the entire mass of added folic acid to be present in our films. For comparison purposes, films were also obtained containing alginate and glycerol (10G/Alg) and alginate, glycerol, and folic acid (10G/Alg/FA), respectively.

#### 2.2.3. Morpho-Structural Characterization of Magnetic Nanoparticles and Magnetic Alginate Film

##### X-ray Diffraction Analysis (XRD)

The phase identification of the Fe_3_O_4_ nanoparticles was assessed by powder X-ray diffraction using a PANalytical Empyrean diffractometer with Cu Kα radiation (λ = 1.541874). Diffractograms were acquired at the 2θ range between 10–80°, with a step size of 0.026⁰. The average crystallite size was determined based on the Debye–Scherrer equation.

##### Vibrating Sample Magnetometry (VSM)

Both the Fe_3_O_4_ nanoparticles and the magnetic film were subjected to magnetic measurements using a Lakeshore Gauss 7400 VSM (Westerville, OH, USA) by applying a magnetic field between −10 kOe and +10 kOe at room temperature.

##### Fourier Transformed Infrared Spectroscopy and Microscopy

Infrared spectra of the 10G/Alg, 10G/Alg/FA, 10G/Alg/FA/Fe_3_O_4_, and the constituent materials (sodium alginate, folic acid, glycerol) were recorded with an iS50 FT-IR spectrometer (Thermo Fischer Scientific, Waltham, MA, USA) in ATR mode. Samples were scanned in the range between 4000 and 400 cm^−1^ at 4 cm^−1^ resolution.

The FT-IR maps were assessed with a Nicolet iS50R FTIR microscope with a DTGS detector (Thermo Fisher Scientific, Waltham, MA, USA) to identify the structural homogeneity of the magnetic films.

##### Scanning Electron Microscopy (SEM)

The surface and cross-section of the magnetic alginate film were evaluated using scanning electron microscopy (FEI Inspect F50, Eindhoven, The Netherlands) after coating the film with a thin gold layer using a sputtering coater. SEM images were acquired operating at 30 KeV at different magnifications.

##### Thermogravimetry and Differential Scanning Calorimetry (TG-DSC)

To investigate the thermal stability of the film, a sample was placed in an alumina crucible and subjected to thermal treatment using a STA TG/DSC Netzsch Jupiter 449 F3 equipment (Selb, Germany). Thermogravimetric analysis was performed between room temperature and 900 °C in a dynamic atmosphere.

##### Folding Endurance

Folding endurance is expressed as the number of folds required to break the film, giving an indication of film brittleness. It was manually measured for three different 2 × 2 cm specimens of the obtained film by repeatedly folding the specimens in the same place until the film broke. The experiment was carried out in triplicate.

##### Weight and Thickness Uniformity

The film was cut into 10 circular pieces of approximately 5 mm from different areas of the cast film using a hole puncher. The weight uniformity was evaluated by weighing all the pieces on an analytical balance (Mettler Toledo New Classic MS205DU/M, Mettler Toledo, Greifensee, Switzerland), and the thickness was measured using a digital caliper.

##### Percentage Moisture Loss (%)

The specimens used for assessing weight and thickness uniformity were left to air-dry at room temperature, and their weight was recorded again after 24 h. The moisture percentage was then determined by dividing the initial weight by the final weight.

##### pH Determination

Since an acidic or alkaline pH can dramatically affect the biocompatibility of a device, the pH was determined after suspending the ten specimens in water for two days.

##### Drug Uniformity

Five specimens collected from different areas of the film were sampled using the same hole puncher as above and dissolved in 10 mL of EDTA (7.5 g/L) using an ultrasonic bath (Bandelin Sonorex Digitec, Berlin, Germany). After the films were completely broken down, they were diluted and evaluated for absorbance with an UV-Vis spectrophotometer between 300 and 450 nm.

#### 2.2.4. Folic Acid Release Assay and Release Kinetics Evaluation

**Figure 1:**
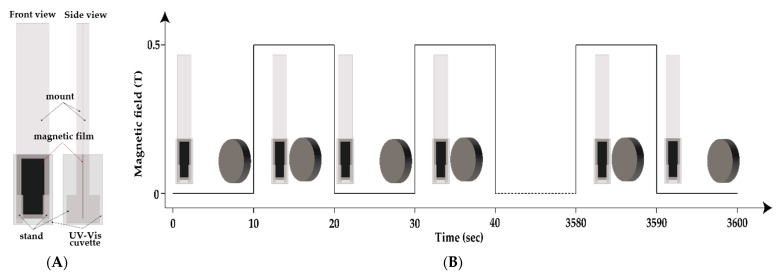
(**A**) PMMA mount placed in a UV-Vis cuvette. (**B**) Experimental set-up used to study the outcome of applying a magnetic field in triggering folic acid release at pH = 8 via a magneto-mechanical trigger. The digital signal representation corresponds to the switching state of the magnetic field, where the high peaks indicate the magnetic field is on, while the low peaks stand for the off state of the magnetic field.

Release studies were performed using two magnetic film pieces with similar weights. Specifically, after cross-linking, the magnetic film (10G/Alg/FA/Fe_3_O_4_) was cut into two different rectangular pieces (10 mm × 20 mm × 0.32 mm) and placed in customized in-house poly(methyl methacrylate) (PMMA) mounts manufactured using a CO_2_ laser-cut machine. The mounts contained a slot of 7 × 20 mm and two additional supports for the lower part, which ensured both support for the mount and a constant pressure on the lower side of the films. The film was fixed inside the mounts so that a constant pressure was applied on all four sides of the films, while exposure to the release medium was achieved through the slot. The total width of the mount was designed so that it fitted a 1 cm quartz cell in order to directly assess UV-Vis measurements for the release environment (Figure 1A). Release studies were conducted in a 2 mL alkaline environment (double distilled water, pH = 8) at room temperature. The sample that was not exposed to the magnetic field is further mentioned as the reference. Exposure to the magnetic field was carried out manually. Precisely, the release study involved repeated magnetic field induction and removal steps of 10 s each by joining the NdFeB magnet (0.5 T) with the cuvette and pulling it 20 cm away, respectively. A schematic illustration of the experiment is presented in Figure 1B.

The total data collection time was set to 60 min. The actual measurements were performed using an Evolution 300 double-beam spectrophotometer (Thermo Fisher Scientific, Waltham, MA, USA). Spectra of the release media were recorded in the 300–450 nm wavelength range at pre-set time intervals of 5, 10, 20, 30, 40, 50, and 60 min, respectively. Folic acid quantification was performed using a six-point calibration curve covering a 5–30 µg/mL range. The relationship between absorbance and concentration proved to be linear for the selected working range, at a 367 nm wavelength, with a correlation coefficient (R^2^) of 0.998. A solution of 15 µg/mL was used to confirm the intra-day precision of the method, while the accuracy was determined by analyzing the recovery after spiking the solvent (water, pH = 8) with a known concentration of folic acid (mean concentration of the calibration curve). The final drug release percent was calculated using Equation (1), where the concentration was determined based on the Beer–Lambert Law.

(1) $$Drug\;release\;\left(\%\right)=\frac{Concentration\;\left[\mu g/mL\right]\cdot Volume\;of\;medium\;\left[mL\right]}{Total\;amount\;of\;folic\;acid\;\left[\mu g\right]}\cdot 100$$

The experimental data were further fitted using the power law model, a semi-empirical mathematical model (Equation (2)):

(2) $$\frac{M_{t}}{M_{\infty }}=K\cdot t^{n}$$

where *M_t_*/*M*_∞_ is the amount of drug released at time *t*, *K* is the release constant, and *n* is the diffusion exponent, which describes the transport mechanism.

## 3. Results and Discussions

**Figure 2:**
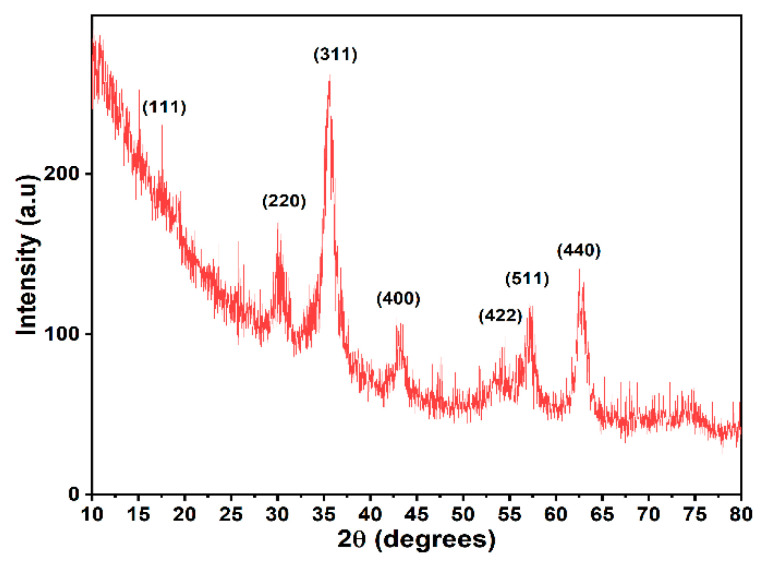
X-ray diffraction pattern of as-synthesized Fe_3_O_4_.

XRD was used for the phase identification of the as-obtained magnetic nanoparticles (Figure 2). The diffractogram revealed diffraction peaks at 2θ = 18.38°, 30.20°, 35.56°, 43.20°, 53.58°, 57.11°, and 62.71° corresponding to the (111), (220), (311), (400), (422), (511), and (440) planes of the cubic Fe_3_O_4,_ Fd-3m space group [46]. All diffraction peaks were in excellent agreement with PDF file no. 96-900-5839. The average crystallite size was calculated using the Debye–Scherrer equation, d = Kλ/(βcosθ), and was approximately 5.22 nm. XRD analysis matches other studies where co-precipitation was used as the synthesis method. However, the crystallite size was much lower than in studies where the co-precipitation was carried out at temperatures higher than room temperature [46,47,48].

The formulated film was visually inspected and can be described as a brown, soft, and non-sticky thick film with a folding endurance of 204 ± 5. The literature does not provide relevant data for similar systems. However, the folding endurance test involved a much higher amplitude compared to the stress applied in the release experiments. The release carried out in this study cumulates about 180 magnetic actuations and thus the obtained value for the folding endurance was suitable for at least one release cycle. The weight and thickness uniformity of the film are shown in Table 1. The film weight (mg) for the ten specimens, labeled from S1 to S10, ranged between 7.6 and 9.6 mg, while the film thickness ranged from 0.30 to 0.39 mm. After drying the samples in air, an average of 62.3 ± 2.52% mass reduction was achieved. The results for pH measurements indicate values between 6.47 and 7.25, which are very good values for future application in the human body.

**Figure 3:**
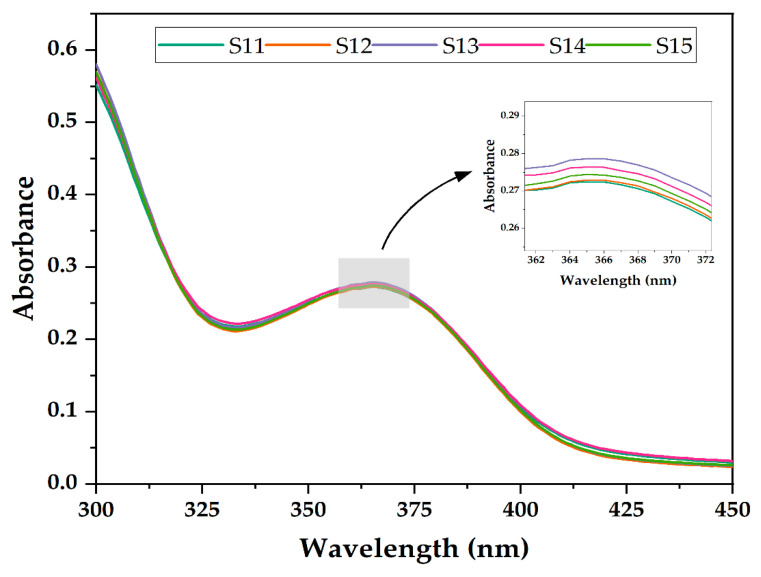
UV-Vis spectra of dissolved specimens of 10G/Alg/FA/Fe_3_O_4_. Insert: enlarged area of the spectra.

To examine the uniformity of the active substance in the mass of the 10G/Alg/FA/Fe_3_O_4_ film, five samples were cut from different areas of the film (marked S11 to S15) and dissolved in EDTA. The UV-Vis spectra obtained for the five specimens are shown in Figure 3. The recorded absorbance ranged from 0.272 to 0.278, indicating a good uniformity in terms of the folic acid throughout the film.

**Figure 4:**
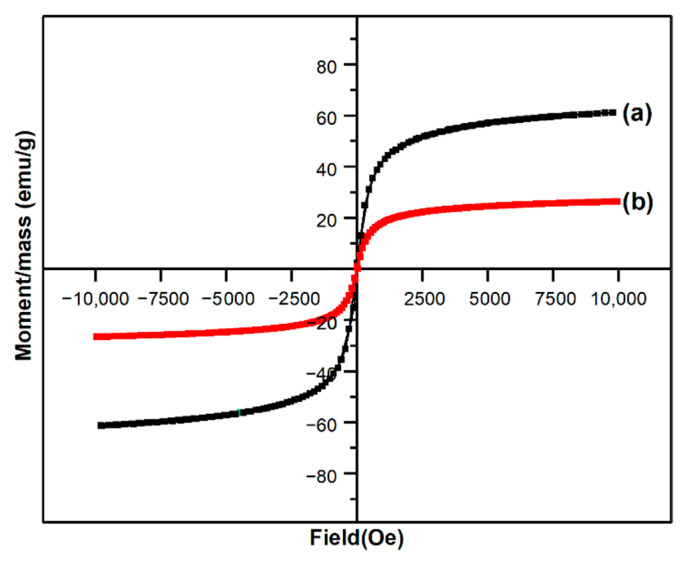
M vs. H curves for pristine Fe_3_O_4_ (a) and 10G/Alg/FA/Fe_3_O_4_ film (b).

Typical properties such as magnetic saturation (M_s_), magnetic remanence (M_r_), and coercivity (H_c_) were extrapolated from the obtained hysteresis curves (Figure 4) for the magnetic particles and the magnetic film. M vs. H curves resulted in sigmoid shapes with almost no coercivity, which is representative for a monomodal system with particle sizes smaller than 20 nm. The lack of hysteresis loop is also an indicator of superparamagnetic behavior [49]. As was indicated by VSM analysis, the obtained magnetic powder had a magnetization of 61.25 emu∙g^−1^ and agrees with other studies where magnetite was obtained by the co-precipitation method [50]. When comparing the M–H curves, there was a noticeable decrease in saturation magnetization when our composite film was investigated. The magnetization decreased 2.31 times, and this is attributed to the fact that the polymeric matrix embraces the particles and behaves like a shield for them. This reduction in magnetization is characteristic of different types of composites embedding iron oxide nanoparticles in polymeric matrixes, such as biopolymers [51,52] or synthetic polymers [53,54].

**Figure 5:**
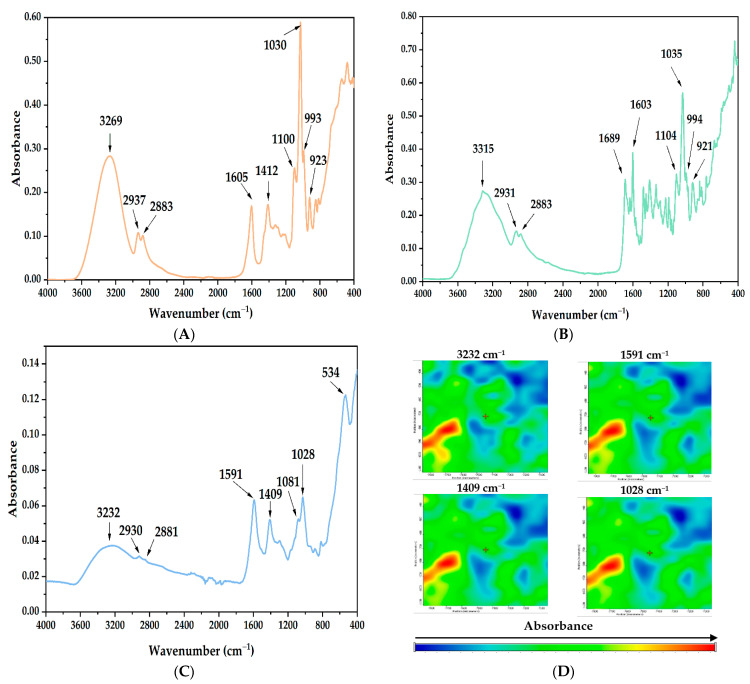
FT-IR spectra of (**A**) 10G/Alg film, (**B**) 10G/Alg/FA film, and (**C**) 10G/Alg/FA/Fe_3_O_4_ film and (**D**) FT-IR maps for 10G/Alg/FA/Fe_3_O_4_ at selected wavenumbers.

The homogeneity of the magnetic film containing folic acid was assessed by FT-IR spectroscopy and microscopy (Figure 5). To confirm that the folic acid and the Fe_3_O_4_ nanoparticles were evenly distributed across the film, IR spectra (Figure 5A–C) were recorded to identify and compare functional groups present in 10G/Alg, 10G/Alg/FA, and 10G/Alg/FA/Fe_3_O_4_ films. As can be seen for the simple 10G/Alg film (Figure 5A), the broad peak between 3600 and 3000 cm^−1^ corresponds to the O–H bond. The band appears to be sharper than usual due to the high content of hydroxyl groups present in glycerol [45]. The two small bands at 2937 cm^−1^ and 2883 cm^−1^ are attributed to ν(C-H) asymmetric and symmetric stretching vibrations. Moving to lower wavenumbers, we encounter two strong peaks at 1605 cm^−1^ and 1412 cm^−1^, corresponding to the stretching vibrations of the COO^−^ functional group present in the alginate structure [55]. When comparing the spectra with the raw materials (Supplementary Materials, Figure S1.), it is noteworthy that the 10G/Alg film has a similar pattern to sodium alginate powder. However, the absorption bands characteristic of the stretching vibration of COO^−^ (1594 cm^−1^) and C-O-C (1025 cm^−1^), respectively, are slightly shifted to higher frequencies, most likely due to the presence of glycerol. Gao and coworkers [56] showed similar behavior, with the band moving to higher wavenumbers with an increasing glycerol content in alginate films. This behavior is explained based on the increased affinity of glycerol for water molecules, weakening the hydrogen bonds between alginate and water and leading to the appearance of hydrogen bonds between glycerol and alginate. In Figure 5B, after mixing the 10G/Alg solution with folic acid (FA), we can see how the region between 1800 cm^−1^ and 1200 cm^−1^ is similar to the FA spectra from Figure S1. The strong peaks between 1689 and 1603 cm^−1^ are correlated to carboxylic groups present in folic acid, and by comparing the spectrum of folic acid with the spectrum of the film containing folic acid, no difference is observed for the specific peaks of the carboxylic groups. This indicates the encapsulation of the active substance in the polymeric mass by chemical interactions rather than by physical entrapment. Finally, by adding the magnetic nanoparticles in the 10G/Alg/FA membrane, a new absorption band appeared at 534 cm^−1^, specific to Fe–O bond vibrations [57]. The peak assignment for the Fe–O bond is also confirmed by the spectrum of the synthesized magnetite (Figure S2, Supplementary Materials). Additionally, the main absorption bands characteristic of 10G/Alg slightly shifted to lower frequencies. The shifts corresponding to carboxyl groups (1591 and 1409 cm^−1^) can be considered as an effect of molecular interaction taking place between the magnetic nanoparticles and the alginate matrix [58,59], while the peaks corresponding to C-H stretching vibrations remain unchanged.

Starting from the FT-IR spectrum, we identified the most intense absorption bands as being at 3232, 1591, 1409, and 1028 cm^−1^, so we selected these for mapping analysis. As we can see from Figure 5D, there are no significant differences between the maps recorded for the selected wavelengths, indicating that the obtained film was structurally homogenous.

**Figure 6:**
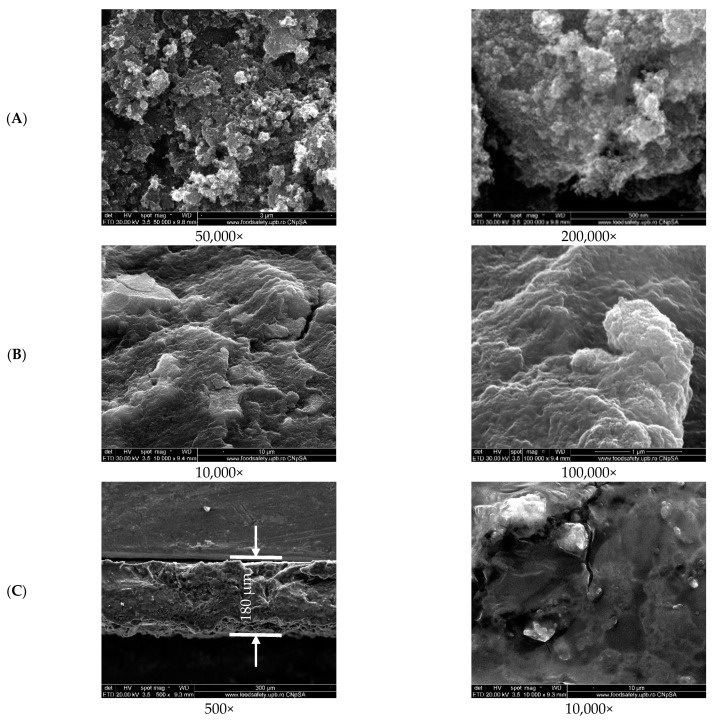
SEM images of magnetic nanoparticles (**A**), surface of magnetic alginate films (**B**), and cross-section of the magnetic alginate film (**C**) at different magnifications.

The SEM images in Figure 6A show the fine magnetic powder obtained at different magnifications, characterized by an agglomeration of nanosized and quasi-spherical shaped particles, which was expected as the material was obtained by co-precipitation [57]. Using the ImageJ processing tool, the particle size and size distribution of the fine magnetic powder were measured. The mean particle size was estimated to be 13.9 ± 1.5 nm, while the size distribution was monomodal, as seen in the Figure S1. The measurements were assessed for 50 particles from different areas of the material.

**Figure 7:**
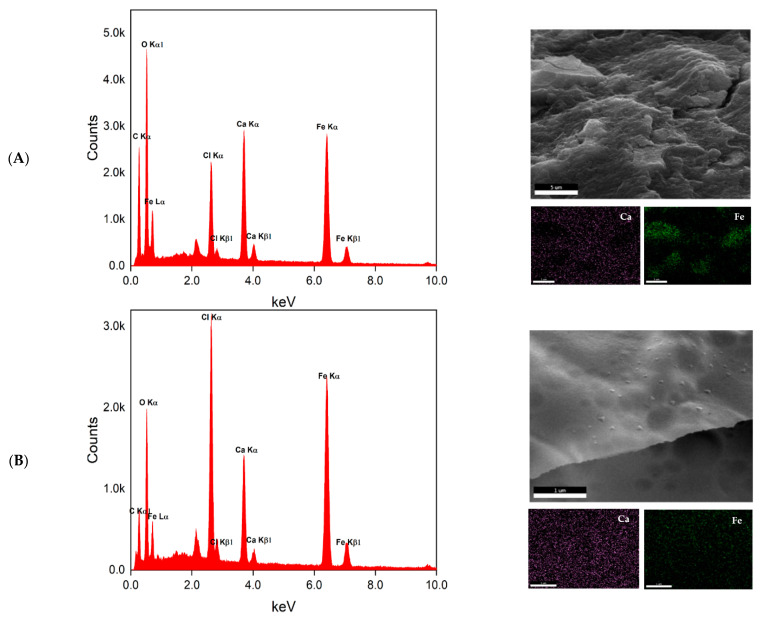
EDAX measurements and mapping for surface (**A**) and cross-section (**B**) of the 10G/Alg/FA/Fe_3_O_4_ film.

Both the surface and cross-section images of the 10G/Alg/FA/Fe_3_O_4_ film (Figure 6B,C) show that a compact material was obtained. The appearance of ravines is most likely caused by the casting method, as it correlates with other studies where casting was employed as a film-making procedure. Furthermore, surface cracks are observed on both the surfaces. The cracks may have been caused by the drying process [60]. The film thickness is approximately 180 µm for the dried film, which agrees with our measurement on the wet film given the fact that after drying the film loses about 62% of the mass. Additional EDS measurements and mapping were assessed to determine how the elements of interest are distributed on the top and cross-section of the magnetic film. Sodium alginate was successfully cross-linked with calcium ions, as is suggested by the presence of the chloride peak at 2.6 keV and the calcium peak at 3.7 keV in EDS spectra. The elements Ca and Fe were selected as most important in identifying possible morphological heterogeneities. EDAX mapping (Figure 7) revealed a uniform distribution of calcium in the structure of the film both in terms of surface and cross-section analysis. A slight agglomeration of iron can be observed when examining the surface of our film. Compared with the image for which the mapping was performed, the agglomerations are most likely caused by the ravines formed on the surface due to the fabrication process. Additionally, when we look at the cross-section mapping, the distribution of iron is homogeneous, with these data being in good agreement with the other data.

**Figure 8:**
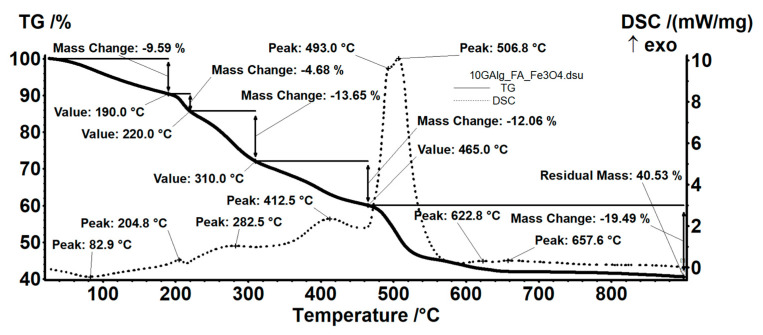
Thermal analysis of room temperature-dried 10G/Alg/FA/Fe_3_O_4_ film.

As deduced from Figure 8, the sample 10G/Alg/FA/Fe_3_O_4_ loses 9.59% of its mass between room temperature and 190 °C. The loss is attributed to water molecules embedded in the polymeric membrane [61], and the DSC curve shows the process is accompanied by an endothermic effect with a minimum at 82.9 °C. At higher temperatures, the sample undergoes a series of degradative-oxidative processes, partially overlapping, accompanied by weakly exothermic effects, which indicates the predominance of oxidation reactions. Alginate degradation begins between 190–220 °C, with fragments breaking away from the polymeric chain [62]. The DSC curve shows a maximum value at 204.8 °C, which is attributed to the oxidation of these fragments. Also, in this interval, the oxidation of Fe^2+^ to Fe^3+^ takes place, transforming magnetite to maghemite [41]. Glycerol oxidation occurs between 220–310 °C, while alginate partial oxidation continues. Folic acid degradation also occurs during this time. The process is accompanied by a weak exothermic effect, showing a broad peak with a maximum at 282.5 °C. In the temperature interval between 310 and 465 °C, the oxidation of the rest of the polymeric backbone continues, accompanied by an exothermic effect at 412.5 °C. The carbonaceous residue is burned after 465 °C, when a mass loss of 19.49% and a strong, asymmetric exothermic effect is recorded, with maximum values at 493 °C and 506.8 °C. The thermal, weakly exothermic effect at 657.6 °C can be attributed to the partial phase transformation of maghemite into hematite [63]. The residue (40.53%) consists of hematite, which is strongly correlated with our magnetite–alginate mass ratio.

**Figure 9:**
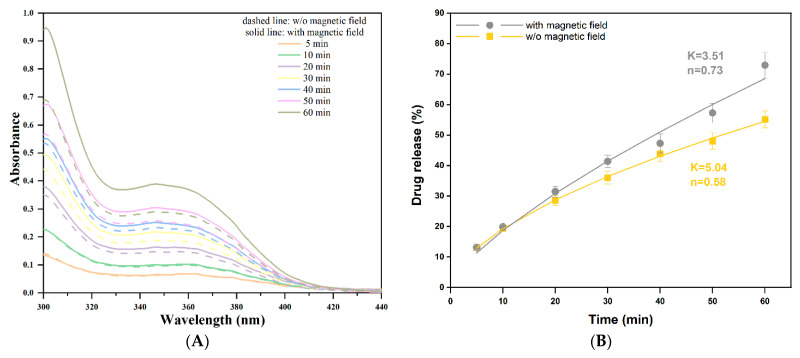
(**A**) Absorbance profile of 10G/Alg/FA/Fe_3_O_4_ films at different time intervals without using a magnetic field (dashed line) and using a 0.5 T magnetic field as a trigger (solid line) and (**B**) folic acid release profile expressed as cumulative drug released. Data expressed as mean ± SD (*n* = 3). The points represent experimental data, and the lines represent fitting results.

In Figure 9, the UV-Vis spectra of the solutions where the 10G/Alg/FA/Fe_3_O_4_ film was immersed, both in the presence and in the absence of the magnetic field, are presented. Both the sample exposed to the magnetic field (solid line) and the sample not exposed to the magnetic field (dotted line) showed an increase in the absorbance unit depending on the exposure time in the release medium. Additionally, Figure 9B shows that both samples have a similar trend towards kinetic release. In the first 5 min, it can see that the difference in absorbance is almost non-existent, which can be explained by the fact that the film needs a certain time to swell and start the release. However, after 10 min, the differences in absorbance values become more visible, as we can see from the graph (Figure 9A), reaching a maximum difference after one hour. Within the selected range (5–30 μg/mL), the Beer–Lambert Law is followed, with an excellent determination coefficient (R^2^) of 0.998. In addition, the precision and accuracy of the method were evaluated, as given in Tables S1 and S2 (Supplementary Materials).

Overall, the sample exposed to the magnetic field released an average of 72.91 ± 3.99% of the available folic acid, while the sample not exposed to the magnetic field yielded an average of 55.10 ± 2.75% of folic acid released within the one-hour timeframe. Our results are similar to [64], where insulin delivery was highly improved by applying an alternative magnetic field to iron oxide nanoparticles embedded in alginate/chitosan beads. It is worth mentioning the authors observed the same time-dependent increase in delivery rate. Levodopa was released from magnetic hydrogels by applying a 0.4 T electromagnetic field [65].

To investigate the drug release from the films, Equation (2) was fitted using the Solver Add-in in Microsoft Excel for non-linear curve-fitting. Figure 9B shows the experimental data points from three replicates of the release experiment and the fitting results of the samples. The sample not exposed to the magnetic field had a diffusion exponent value closer to 0.5, which may indicate a predominant diffusion mechanism. On the other hand, the sample exposed to the alternative magnetic fields suggested a more anomalous release pathway at the level of the alginate magnetic field, with *n* = 0.73, indicating that the process is also guided by other behaviors such as the swelling or erosion of the polymer.

Based on the release data, the magnetic films can be efficient DDSs for specific drugs. Moreover, the application of the intermittent magnetic field (0.5 T for 10 s followed by a relaxation for an additional 10 s) can be exploited to enhance the release. The mechanism of drug delivery can be explained considering the mechanical stress generated by pressure once the membrane is magnetically deflected (the membrane is elongated and also becomes narrower). In this case, the liquid from the inside of the membrane is expulsed and along with it so is the dissolved drug. Once the magnetic force is removed, the membrane reverts to its initial state and absorbs water and a further amount of drug is dissolved, with it being released when magnetic actuation occurs. Considering this complex, indirect mechanism, the release can be considered to be magnetically induced, but the triggering factor is of a mechanical nature. Thus, an overall magneto-mechanical triggering effect can be considered.

## 4. Conclusions

The study aimed to demonstrate that using an external magnetic field could trigger drug release from a magnetic thick film. The release experiment was carried out by immersing the magnetic thick film immobilized in a home-made support directly in the UV-Vis cuvette and alternatively applying a magnet of 0.5 T (10 s followed by a relaxation of 10 s) for a period of up to 1 h. The FT-IR spectra of the films showed that all three components of the system were successfully incorporated into the films, and, based on the shifts of some specific peaks in terms of alginate and magnetite, it can conclude that interactions occur between them. FT-IR microscopy and SEM/EDAX analysis confirmed that a morphologically and structurally homogeneous film was obtained. The UV-Vis measurements demonstrated that the magneto-mechanically triggered sample releases the active substance at a faster rate. The cumulative drug release achieved in 60 min was 72.91 ±3.99% for the magnetic film exposed to the alternating magnetic field, which was higher that of the cumulative drug release associated with the sample not exposed to the magnetic field (55.10 ± 2.75%). The resulting data show the enormous potential of using the magnetic films in BioMEMS controlled release systems based on micropumps. Further studies will to be focused on the evaluation of the influence of the un-uniformities (magnetite agglomeration) over the mechanical stability of the membranes, the hydrophilic/hydrophobic ratio of the membrane materials, and the influence of the porosity of the membranes, etc. The integration of several components in a micropump, such as bonding the film to a drug containing reservoir, would allow for the possibility that the membrane could load itself over time with the drug solution. In this way, it would be possible to release controlled doses of the drug directly to the site of action.

## Figures and Tables

**Table 1:** Determination of thickness, weight uniformity, and percent moisture loss.

	S1	S2	S3	S4	S5	S6	S7	S8	S9	S10	Mean	St. Dev.
Thickness (mm)	0.39	0.32	0.37	0.35	0.36	0.34	0.31	0.31	0.30	0.30	0.34	0.03
Initial weight (mg)	7.6	7.8	8.6	8.6	8.1	9.6	8.4	8.4	7.8	8.3	8.32	0.54
Final weight (mg)	2.6	2.8	3.1	3.1	3.3	3.6	3.6	3.3	2.8	3.2	3.14	0.32
%Moisture	65.8	64.1	64.0	64.0	59.3	62.5	57.1	60.7	64.1	61.4	62.3	2.52
pH	6.47	6.98	7.14	6.82	7.21	7.19	7.24	6.58	7.15	6.95	6.97	0.26

## Data Availability

Not applicable.

## Supplementary Materials

The following supporting information can be downloaded at: https://www.mdpi.com/article/10.3390/nano12203598/s1, Figure S1: Infrared spectra of raw materials: (a) sodium alginate, (b) glycerol, (c) folic acid, and (d) Fe_3_O_4_ nanoparticles; Figure S2: Particle size statistic results for bulk magnetite nanoparticles; Figure S3: UV-Vis calibration curve of folic acid at 367 nm; Table S1: Precision study of folic acid at 367 nm; Table S2: Accuracy study for a spiked solution of folic acid.
