# Supplementary material for: Magneto-Mechanically Triggered Thick Films for Drug Delivery Micropumps
Source: Nanomaterials (Basel). 2022 Oct 13;12(20):3598. doi: 10.3390/nano12203598 (PMC9607447; doi:10.3390/nano12203598)
Supplement: Supplementary file 1 [file nanomaterials-12-03598-s001.zip › nanomaterials-1915770-supplementary.pdf]

nanomaterials

*Supplementary Material*

# Magneto-Mechanically Triggered Thick Films for Drug Delivery Micropumps

**Georgiana Dolete [1,2], Cristina Chircov [1,2], Ludmila Motelica [1,2], Denisa Ficai [2,3,*], Ovidiu-Cristian Oprea [2,3,4], Marin Gheorghe [5,6], Anton Ficai [1,2,4] and Ecaterina Andronescu [1,2,4]**

1 Department of Science and Engineering of Oxide Materials and Nanomaterials, Faculty of Chemical Engineering and Biotechnologies, University POLITEHNICA of Bucharest, Gh. Polizu 1-7, 011061 Bucharest, Romania
2 National Center for Micro and Nanomaterials, University POLITEHNICA of Bucharest, Splaiul Independentei 313, 060042 Bucharest, Romania
3 Department of Inorganic Chemistry, Physical Chemistry, and Electrochemistry, Faculty of Chemical Engineering and Biotechnologies, University POLITEHNICA of Bucharest, Gh. Polizu 1-7, 011061 Bucharest, Romania
4 Academy of Romanian Scientists, Ilfov Street 3, 050044 Bucharest, Romania
5 SC NANOMEMS SRL, George Coșbuc 9, 505400, Râșnov, Romania
6 Center for Technological Electronics and Interconnection Techniques, University Politehnica of Bucharest, Bulevardul Iuliu Maniu, 061071 Bucharest, Romania
* Correspondence: denisaficai@yahoo.ro

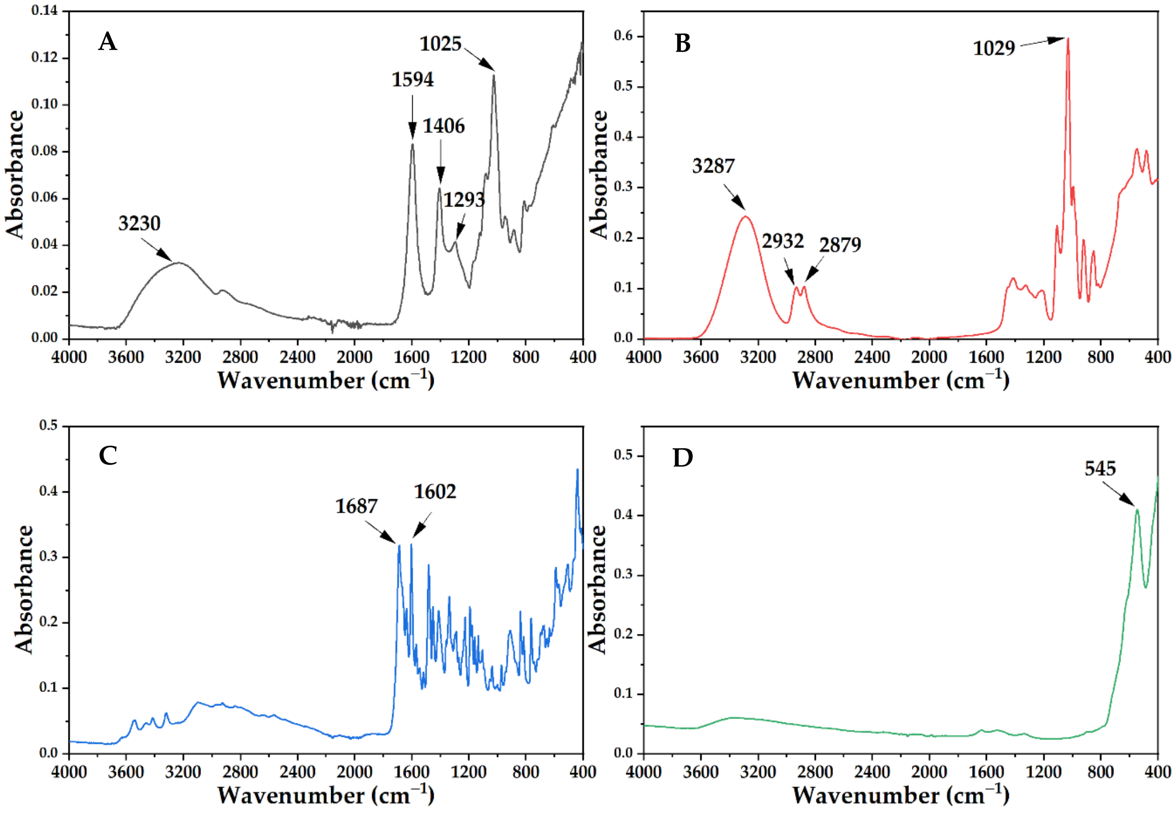


**Figure S1.** Infrared spectra of raw materials: (**A**) sodium alginate, (**B**) glycerol, (**C**) folic acid, and (**D**) $Fe_3O_4$ nanoparticles.

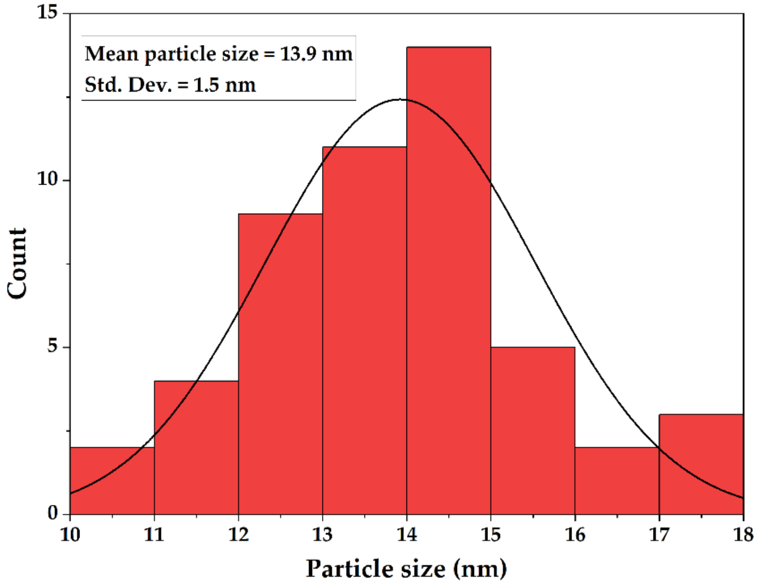


**Figure S2.** Particle size statistic results for bulk magnetite nanoparticles.

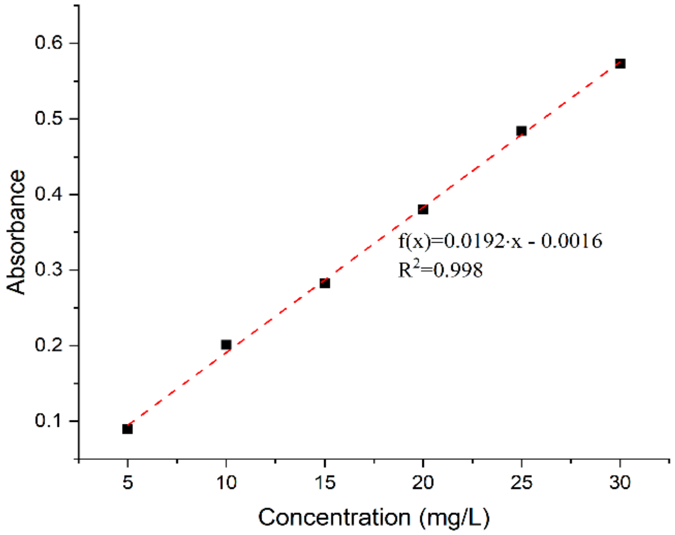


**Figure S3.** UV-Vis calibration curve of folic acid at 367 nm.

**Table S1.** Precision study of folic acid at 367 nm.

| Sample No. | Absorbance |
|---|---|
| 1 | 0.283 |
| 2 | 0.283 |
| 3 | 0.281 |
| 4 | 0.281 |
| 5 | 0.282 |
| **Mean** | **0.282** |
| **±SD** | **0.001** |
| **%RSD** | **0.378** |

nanomaterials

**Table S2.** Accuracy study for a spiked solution of folic acid.

| Theoretic concentration (µg/ml) | Absorbance | Calculated Concentration (µg/ml)[1] | %Recovery[2] |
|---|---|---|---|
| 17.5 | 0.329 | 17.2 | 98.39 |

[1]Calculated based on the obtained linear equation f(x)=0.0192x-0.0016.
[2]%Recovery = (Calculated concentration/Theoretic concentration) x 100.
